# Identification of Clinical Features Associated with Mortality in COVID-19 Patients

**DOI:** 10.1007/s43069-022-00191-3

**Published:** 2023-03-04

**Authors:** Rahimeh Eskandarian, Roohallah Alizadehsani, Mohaddeseh Behjati, Mehrdad Zahmatkesh, Zahra Alizadeh Sani, Azadeh Haddadi, Kourosh Kakhi, Mohamad Roshanzamir, Afshin Shoeibi, Sadiq Hussain, Fahime Khozeimeh, Mohammad Tayarani Darbandy, Javad Hassannataj Joloudari, Reza Lashgari, Abbas Khosravi, Saeid Nahavandi, Sheikh Mohammed Shariful Islam

**Affiliations:** 1 Internal Medicine Research Center, Semnan University of Medical Sciences, Semnan, Iran; 2 Institute for Intelligent Systems Research and Innovation (IISRI), Deakin University, Victoria, Australia; 3 Rajaei Cardiovascular Medical and Research Center, Iran University of Medical Sciences, Tehran, Iran; 4 Omid Hospital, Iran University of Medical Sciences, Tehran, Iran; 5 Department of Biology, Faculty of Basic Sciences, Shahrekord Branch, Islamic Azad University, Shahrekord, Iran; 6 Department of Computer Engineering, Faculty of Engineering, Fasa University, Fasa, 74617-81189 Iran; 7 Data Science and Computational Intelligence Institute, University of Granada, Granada, Spain; 8 System Administrator, Dibrugarh University, Assam, 786004 India; 9 School of Architecture, Islamic Azad University Taft, Taft, 8991985495 Iran; 10 Department of Computer Engineering, Faculty of Engineering, University of Birjand, Birjand, Iran; 11 Department of Computer Engineering, Amol Institute of Higher Education, Amol, Iran; 12 Institute of Medical Science and Technology, Shahid Beheshti University, Tehran, Iran; 13 School of Engineering and Applied Sciences, Harvard Paulson, Harvard University, Allston, MA 02134 USA; 14 Institute for Physical Activity and Nutrition, School of Exercise and Nutrition Sciences, Deakin University, Geelong, VIC 3220 Australia; 15 Cardiovascular Division, The George Institute for Global Health, Newtown, Australia; 16 Sydney Medical School, University of Sydney, Camperdown, Australia

**Keywords:** COVID‐19, Mortality, Risk factors, Symptoms, Machine learning

## Abstract

Understanding clinical features and risk factors associated with COVID-19 mortality is needed to early identify critically ill patients, initiate treatments and prevent mortality. A retrospective study on COVID-19 patients referred to a tertiary hospital in Iran between March and November 2020 was conducted. COVID-19-related mortality and its association with clinical features including headache, chest pain, symptoms on computerized tomography (CT), hospitalization, time to infection, history of neurological disorders, having a single or multiple risk factors, fever, myalgia, dizziness, seizure, abdominal pain, nausea, vomiting, diarrhoea and anorexia were investigated. Based on the investigation outcome, decision tree and dimension reduction algorithms were used to identify the aforementioned risk factors. Of the 3008 patients (mean age 59.3 ± 18.7 years, 44% women) with COVID-19, 373 died. There was a significant association between COVID-19 mortality and old age, headache, chest pain, low respiratory rate, oxygen saturation < 93%, need for a mechanical ventilator, having symptoms on CT, hospitalization, time to infection, neurological disorders, cardiovascular diseases and having a risk factor or multiple risk factors. In contrast, there was no significant association between mortality and gender, fever, myalgia, dizziness, seizure, abdominal pain, nausea, vomiting, diarrhoea and anorexia. Our results might help identify early symptoms related to COVID-19 and better manage patients according to the extracted decision tree. The proposed ML models identified a number of clinical features and risk factors associated with mortality in COVID-19 patients. These models if implemented in a clinical setting might help to early identify patients needing medical attention and care. However, more studies are needed to confirm these findings.

## Introduction

In January 2020, severe acute respiratory syndrome coronavirus 2 (SARS-CoV-2) was discovered [1]. Since then, the virus has spread exponentially and caused immense human suffering worldwide [2–6]. The high number of deaths and the global spread of coronavirus disease (COVID-19) led the World Health Organization to announce it as a pandemic on 12 March 2020 [7, 8]. The world has suffered a high toll from this pandemic regarding increased poverty, economic repercussions and human lives lost to date [9]. A considerable portion of the population is asymptomatic carriers for COVID-19. The most common symptoms include fever (83%), cough (82%) and shortness of breath (31%) [10]. Patients with COVID-19 also demonstrate ground-glass opacity and multiple mottling in patients with pneumonia in chest X-rays.

COVID-19 patients typically yield decreased eosinophils and lymphocyte counts, lower median haemoglobin values, and enhanced neutrophil counts, WBC and serum levels of ALT, AST, LDH and CRP [11]. For severe COVID-19 development, initial CRP serum levels have been considered as an independent predictor [12]. Although the lung is the main target of COVID-19 infection, the widespread distribution of ACE2 receptors in organs [13] may lead to gastrointestinal, liver, kidney, central nervous system, cardiovascular and ocular damage needs to be closely observed [14]. Patients with acute respiratory distress syndrome may deteriorate speedily and die of multiple organ failure [10] induced by the so-called cytokine storm. The severity of COVID-19 is also associated with elevation of D-dimer levels. The elevated D-dimer levels may reflect the risk of disseminated coagulopathy in patients with severe COVID-19, which may require anticoagulant therapy [15].

Early surveillance, contact tracing, testing and strict quarantine strategies have been used by many countries that maintained a low COVID-19 mortality rate [16–18]. Many of these countries had adopted digital technology to implement effective strategies and integrate them with healthcare delivery systems [19–21]. Pandemic plans are thorny to achieve manually but can be facilitated using digital health technology [22–24]. Early flattening of the incidence curve was possible in some countries like South Korea, which had integrated government-coordinated mitigation and containment processes into digital technology [25, 26]. UpCodeto utilized the data generated by the Singapore Ministry of Health to portray infection trends and recovery time [27]. The web-based platform HealthMap and COVID-19 dashboard of Johns Hopkins University provides an up-to-date scenario of COVID-19 deaths and cases across the world [28].

AI algorithms play a vital role in the integration of digital technology with healthcare [29–32]. For example, Shi et al. [33] analysed the characteristics, risk factors and outcomes for in-hospital mortality of COVID-19 patients with diabetes. They abstracted laboratory, clinical and demographic data of the patients and the risk factors associated with mortality were identified by performing multivariable Cox regression analyses. The outcomes of COVID-19 patients with diabetes were lower than age- and gender-matched patients without diabetes. Yadaw et al. [34] devised a useful prediction model of COVID-19 mortality utilizing unbiased computational techniques and detected the most predictive clinical features. Their machine learning (ML) framework was mainly based on three clinical features: minimum oxygen saturation throughout patients’ medical encounters, age and type of patient encounter. Their COVID-19 mortality prediction model exhibited a competitive accuracy. Although a number of studies have explored the association of mortality with clinical features of COVID-19, those studies did not provide a comprehensive list of clinical features associated with COVID-19 mortality. In addition, most of the predictive COVID-19 ML models were based on Chinese data; hence, it might not be relevant in other parts of the world. In this study, we tried to cover these two weaknesses of previous researches. We aimed to determine the set of clinical features associated with COVID-19 mortality in Iranian cases for the first-time using ML approaches.

## Methods

In this section, the data collection process, the employed ML model and conducted statistical tests are presented. C4.5 decision tree is used as the ML model to predict whether a COVID-19 patient survives or not given his/her symptoms and medical conditions.

### Study Settings, Population and Recruitments

We collected medical reports of all COVID-19 patients (*n* = 3008) who have been referred to Semnan hospital in Iran between March 2020 to November 2020. Data on sociodemographic features and clinical factors such as gender, age, number of months of infection and hospitalization, inpatient department, fever, myalgia, seizures and dizziness were investigated to determine their effects on the mortality of COVID-19 patients. All of the investigated features are categorical except age, blood pressure and oxygen saturation which are continuous. The dataset collection process has been done under the direct supervision of registered medical experts. Considering that data collection is error prone, samples with suspicious values were corrected if possible and discarded otherwise.

### ML Models

In this research, C4.5 decision tree [35] is used for classification of patients. The C4.5 algorithm makes decisions using a set of training tree data. To do this, to create each node of the decision tree, C4.5 algorithm selects one of the features of training data that can more effectively partition the training samples. This selection is made based on the concept of entropy. Any attribute that can classify samples into purer categories is selected sooner. Then, the train dataset is categorized according to that attribute, and several branches are created. This process is repeated in each branch. If all the instances in the subcategory belong to a class, a leaf node is created for the decision tree and the class of those instances is specified, but if all the instances do not belong to a class and a new attribute cannot be selected for any reason, C4.5 creates a decision node using the expected value of the class. In addition, some dimension reduction algorithms such as PCA [36], PSL [37] and t-SNE [38] were used to show the samples according to important features. Dimension reduction is one of the major tasks for multivariate analysis. PCA as a linear dimension reduction algorithm is applied without considering the correlation between the dependent and the independent variables. However, PLS is applied based on the correlation. On the other hand, t-SNE algorithm estimates a similarity measure between pairs of samples in the high and the low dimensional spaces.

### Ethics Approval

Local ethical committee of the Semnan University of Medical Sciences approved this research. The patients were informed about this research aims, and written consent was obtained before data collection.

### Statistical and ML Analysis

We analysed the dataset features using MATLAB 2018b software. To determine difference between the two patient groups (i.e. alive and dead), Wilcoxon rank‐sum test [39] and Fisher’s exact test [40] were used for continuous and categorical data, respectively. The statistical significance of the two tests was set to *P* ≤ 0.05. In C4.5, the information gain was employed as the criterion to determine the attributes to be used as tree nodes. At each tree node, the attributes with minimum entropy were selected to form the children of that node. The number of children is equal to the number of possible values that the selected attribute can have. The size of each node ${N}_{i}$ is the number of examples in the sub-tree that has ${N}_{i}$ as its root. Only those nodes were split whose size was greater than or equal to the minimal size for split parameter. In our experiments, the split parameter was set to 4. For C4.5, the size of each leaf node (the number of examples in it) must be set as well. Finally, the last parameter that must be specified is the minimal gain. Only the nodes with gain greater than the minimal gain were considered for split operation. Increasing the minimal gain leads to fewer splits and smaller decision tree.

## Results

**Fig. 1:**
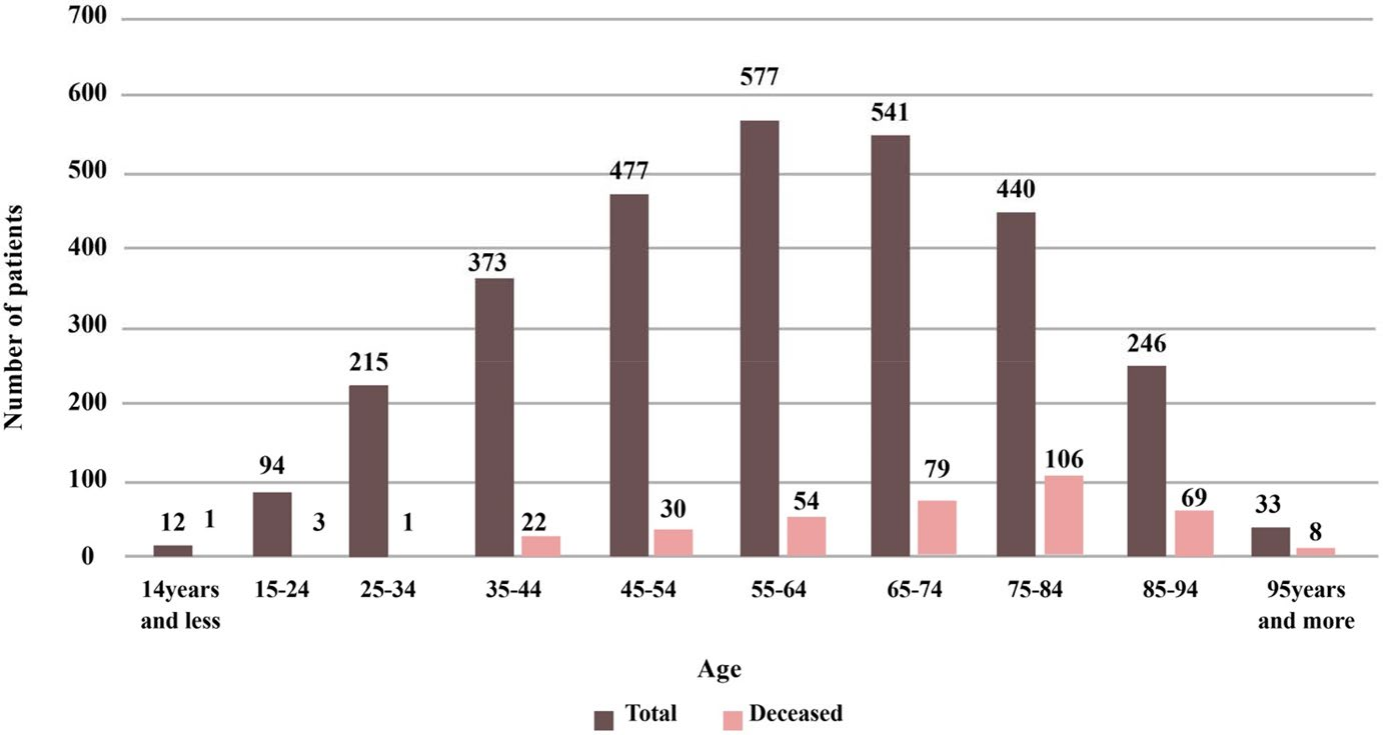
The relationship between COVID-19 mortality and age

Of the 3008 patients with COVID-19, 94.5% (2844) were of Iranian nationality and 5.5% (164 cases) were Afghan nationals. 56% were men, and 44% were women with an age average (± SD) 59.3 ± 18.7 years (1–100 years). In Fig. 1, the histogram of COVID-19 casualties for different age intervals has been shown. Of the patients who were referred to the hospital during this period, 18.5% were required to be admitted to the intensive care unit and the rest to the isolated and normal wards. Three hundred seventy-three of these 3008 cases were deceased. Three hundred eighty-seven patients (12.9%) with COVID-19 were in contact with the infected person, and 2621 patients (87.1%) declared any contact with the infected person. About 70.4% of patients referred to hospital personally, and 653 (21.7%) of them were conveyed to the hospital by pre-hospital emergency, 199 (6.6%) by private ambulance and 38 (1.3%) by ambulances from other centres.

Of the studied patients, 20 patients (0.7%) had a history of previous infection. Patients admitted to the hospital were associated with symptoms including 32.2% fever, 28% cough, 14% myalgia, 43.3% loss of consciousness, 0.8% loss of sense of smell, 0.5% loss of taste, 0.4% seizures, 4.6% headache, 1.6% dizziness, 0.4% paresis, 0.1% plague, 3.8% chest pain, 3.8% chills, 0.5% sweating, 0.5% dry throat and sore throat, 7.8% weakness and lethargy, 0.2% sputum excretion, 0.2% gastrointestinal bleeding, 2.3% abdominal pain, 5.4% Nausea, 3.8% vomiting, 2.9% diarrhoea and 4.4% anorexia. Other initial symptoms included haemoptysis (in 2 patients), oedema, restlessness, delirium, earache, constipation, palpitations, sudden loss of vision and haematuria (each in one case). Fifty cases (1.7%) were a smoker, and 70 cases (2.3%) were addicted to drugs. Two thousand seven hundred sixty-four patients underwent CT scan, of which 2277 had symptoms, and 244 did not undergo CT scan. One hundred seventy-eight patients (5.9%) needed mechanical ventilation at the beginning of the study, and the others did not. The average (± standard deviation) level of oxygen saturation at referral was 89.3% ± 7.4% (39–100%). 37.2% of patients had more than 93% oxygen saturation.

The number of patients’ respiration per minute were also measured in such a way that 0.3% (9 patients) did not breathe at all, 194 patients (6.4%) with 10–14 breaths, 1068 patients (35.5%) had 14–18 breaths, and 1296 patients (43.1%) showed 18–122 breaths per minute. Indeed, 353 patients (11.8%) had 22–28 breaths, and 88 patients (2.9%) had more than 28 breaths per minute. The average (± SD) of patients’ body temperature at the time of referral was 37.1 ± 0 0.7 °C [35–40]. 21.8% of patients had a fever at the time of referral.

The average (± SD) duration of symptoms until referral was 4.7 ± 13.9 days. In these patients, 1670 patients (55.5%) had risk factors or underlying diseases, so that 104 patients (3.5%) had cancer, 16 patients (0.5%) had liver disease, 588 patients (19.5%) with diabetes, 39 (1.3%) with chronic haematological diseases, 15 (0.5%) with immunodeficiency, 586 patients (19.5%) with cardiovascular diseases, 177 patients (5.9%) with kidney diseases, 108 patients (3.6%) with asthma, 99 patients (3.3%) with chronic lung diseases, 127 patients (4.2%) with neurological diseases, 695 patients (23.1%) with hypertension, 26 patients (0.8%) with CVA and stroke, 8 patients (0.2%) with neurosurgery-related problems, 28 patients (0.9%) with hypothyroidism, 43 patients (1.4%) with other neurological diseases, 42 patients (1.3%) with hyperlipidaemia, 15 patients (0.4%) with prostate, 43 patients (1.4%) with psychological diseases, 10 patients (0.3%) with history of veteran chemical warfare and 24 patients (0.7%) had anaemia. Out of 177 patients with kidney disease, 77 were on dialysis.

One thousand three hundred thirty-eight patients (44.5%) had no risk factor and underlying disease. Eight hundred twenty-three (27.4%) and 567 (18.8%) patients had one and two risk factors, respectively. Three and four risk factors were observed in 218 (7.2%) and 52 cases (1.7%), respectively. Nine (0.3%) and one patient (0.05%) had five and six risk factors, respectively. Among 3008 investigated patients, 112 (3.7%) were hospitalized, 2523 (83.9%) were discharged, and also 373 (12.4%) died. The average (± SD) duration of hospitalization was 6.17 ± 6.3 days (1–87 days), of which 236 patients (7.8%) did not need hospitalization, and 2154 patients (71.6%) required 1–7 days of hospitalization. Three hundred seventy-six cases (12.5%) 8–14 days, 137 cases (4.6%) 15–21 days, 59 cases (2%) 22–28 days and 16 cases (0.5%) more than 28 days were hospitalized.

**Fig. 2:**
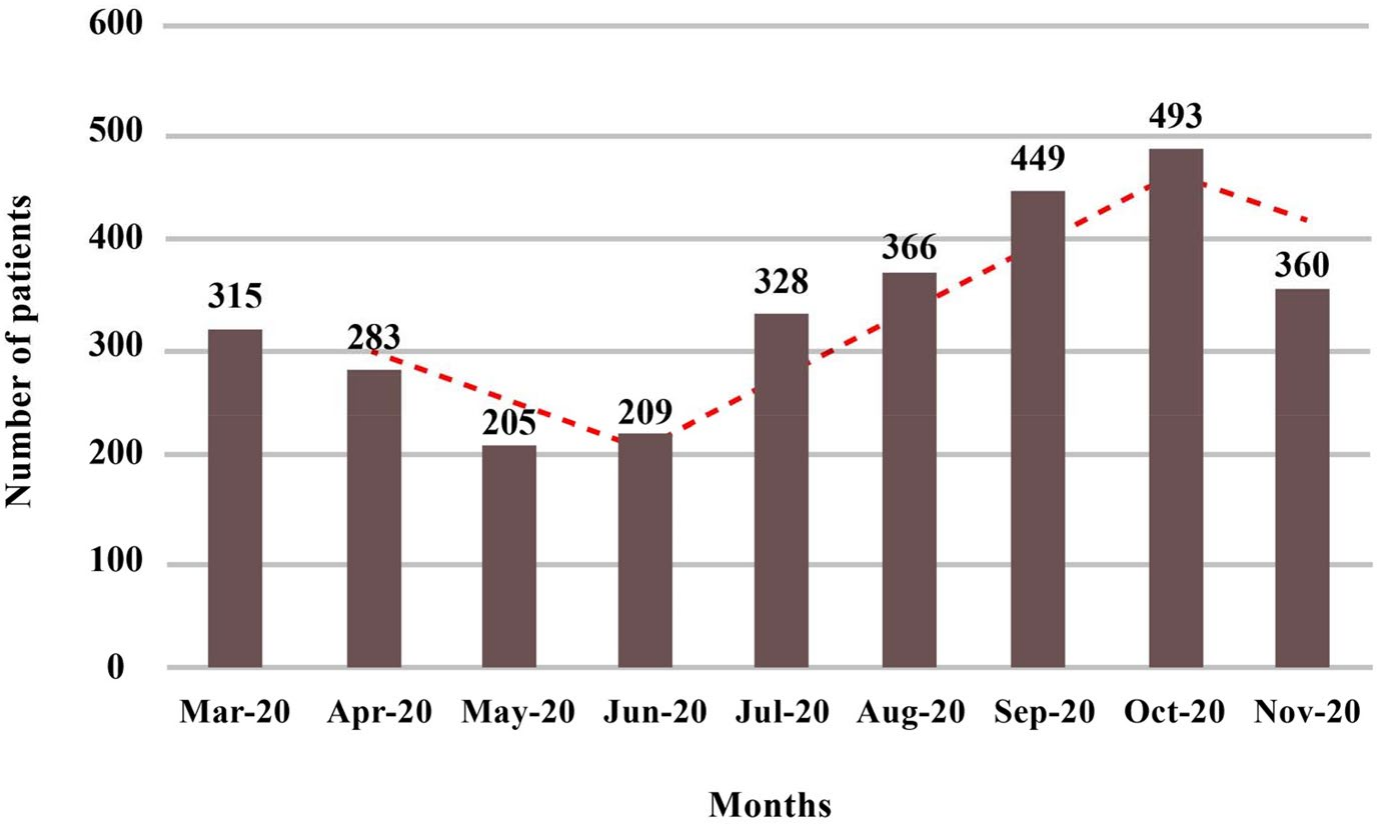
Number of patients between March 2020 and November 2020

According to these data, the prevalence of COVID-19 infection was high in March 2020 and then had the lowest incidence in May and June and finally reached its peak in October and was associated with the fewer incidence in November (Fig. 2).

### The Effect of Early Symptoms on the Outcome of Patients’ Deaths

Table 1 shows the effect of different features on the mortality rate. Mortality was not significantly different between men (1684 cases) and women (1324 cases). There was a significant correlation between mortality and age of patients (*P* < 0.001), infection time (*P* < 0.001) and the hospitalization ward (isolated ward, intensive care unit, normal ward) (*P* < 0.001). Symptoms such as fever, myalgia, dizziness, seizure, abdominal pain, nausea, vomiting, diarrhoea and anorexia were occurred without having mortality related to COVID-19 (*P* > 0.05). There was a significant association between mortality and headache in infected patients (*P* < 0.011). Chest pain was also associated significantly with COVID-19-related mortality (*P* < 0.045). Decreased level of consciousness was also significantly associated with COVID-19-related mortality (*P* < 0.0001). Respiratory distress, oxygen saturation less than 93%, lower respiratory rate and need for mechanical ventilation were associated with COVID-19-related mortality (*P* < 0.004, *P* < 0.001, *P* < 0.001 and *P* < 0.001, respectively).

**Table 1:** The effect of studied features on the mortality rate

	Number		Deceased		Percent		*P* value
Gender							
Male	1684		216		12.8		0.424
Female	1324		157		11.8		
Age category							
14 years and less	12		1		8.3		0.001
15–24	94		3		3.2		
25–34	215		1		0.5		
35–44	373		22		5.9		
45–54	477		30		6.3		
55–64	577		54		9.4		
65–74	541		79		14.6		
75–84	440		106		24.1		
85–94	246		69		28.1		
95 and more	33		8		24.2		
Month (infection – hospitalization)							
March 2020	315		40		12.7		0.001
April 2020	283		52		18.4		
May 2020	205		25		12.2		
June 2020	209		23		11		
July 2020	328		43		12.8		
August 2020	366		45		12.3		
September 2020	449		60		13.4		
October 2020	493		65		13.2		
November 2020	360		20		5.5		
Inpatient department							
Isolated	1554		90		5.8		0.001
Special	555		248		44.7		
Normal	899		35		3.9		
Symptoms							
Fever	969		109		11.2		0.187
Myalgia	422		40		9.4		0.051
Seizures	13		0		0		0.994
Dizziness	42		4		9.5		0.580
Abdominal pain	68		6		8.8		0.365
Nausea	163		20		12.2		0.954
Vomiting	115		19		16.5		0.174
Diarrhoea	87		9		10.3		0.552
Anorexia	113		15		13.2		0.683
Smoking	50		4		8		0.346
Addiction	70		7		10		0.539
Cancer	104		19		18.2		0.067
Liver disease	16		2		12.5		0.990
Diabetes	588		83		14.1		0.160
Chronic blood disease	39		5		12.8		0.936
Receiving immunosuppressive drugs	14		1		7.1		0.556
Pregnancy	373		0		0		0.994
Other chronic diseases	239		34		14.2		0.373
Kidney disease	177		24		13.5		0.630
Asthma	108		20		18.5		0.052
Lung diseases	99		18		18.1		0.078
Cough	843		87		10.3		0.031
Respiratory distress	1301		187		14.3		0.004
Decreased level of consciousness	198		57		28.7		0.001
Headache	137		7		5.1		0.011
Chest pain	114		7		6.1		0.045
Having symptoms on CT scan	2277		313		13.7		0.001
Need for mechanical ventilation	178		90		50.5		0.001
Oxygen saturation	More than 93%	Less than 93%	More than 93%	Less than 93%	More than 93%	Less than 93%	0.001
	1120	1888	53	320	4.7	16.9	
Respiratory rate	644		95		14.7		0.001
Cardiovascular disease	586		89		15.1		0.023
Neurological diseases	127		25		19.6		0.012
Blood pressure	695		109		15.6		0.003
Having a risk factor	1670		234		14		0.003
Having multiple risk factors	847		139		16.4		0.002

**Fig. 3:**
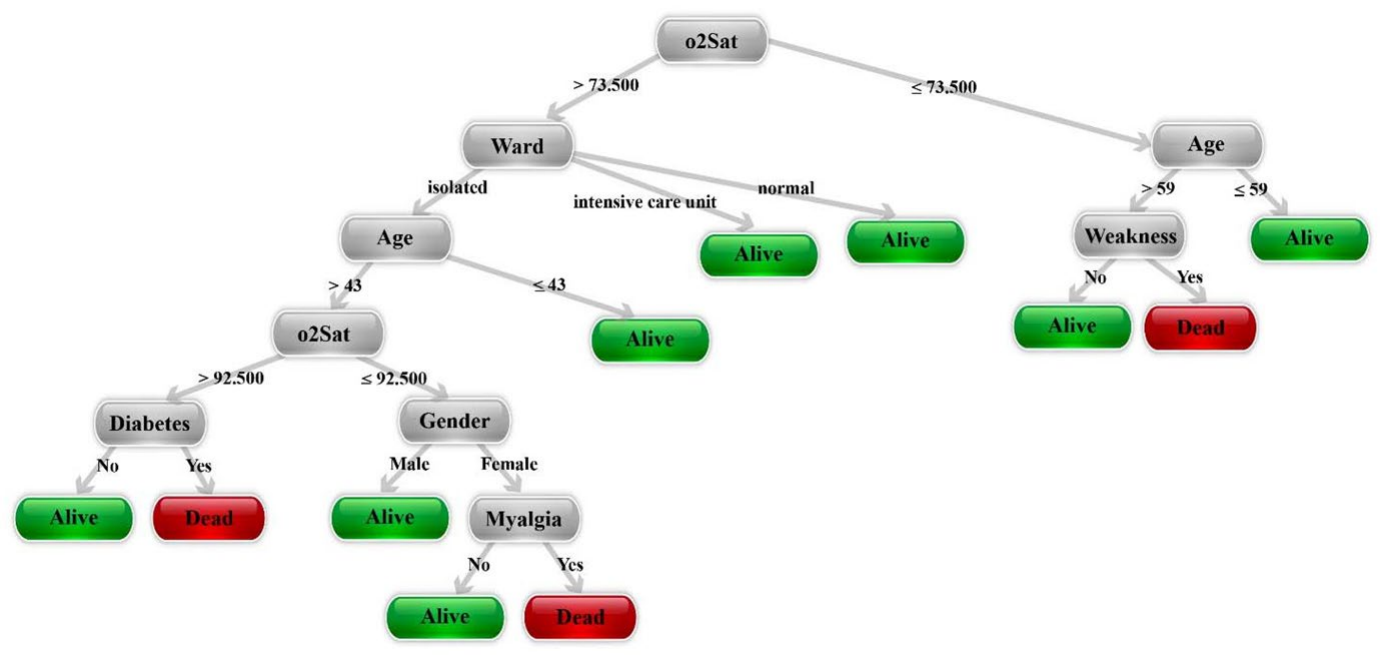
An example of decision tree

**Fig. 4:**
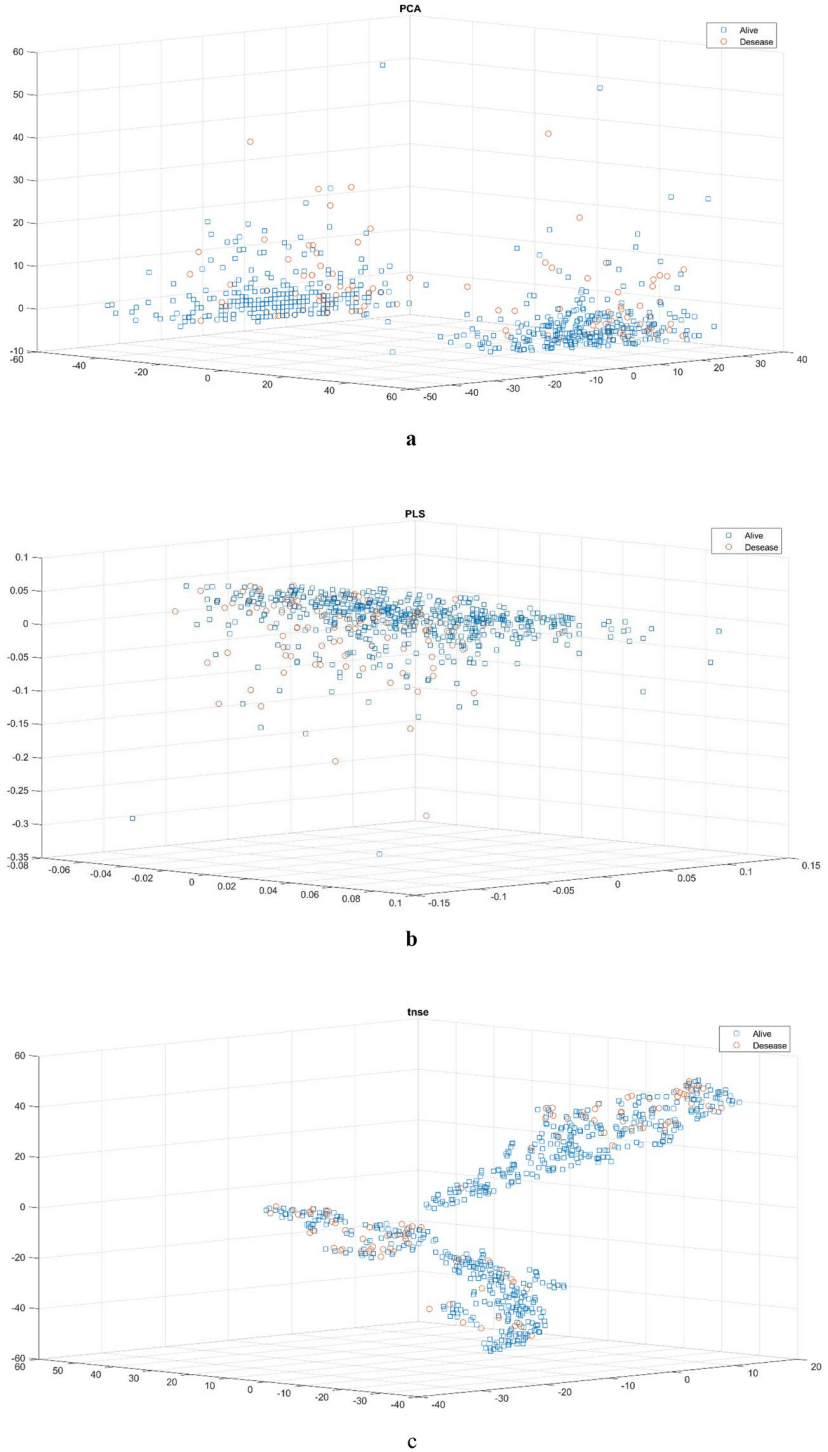
The cases are shown according to their three most important features selected by a PCA, b PLS, and c t-SNE algorithms

Opium addiction, smoking status, pregnancy, diabetes mellitus, underlying cancer, liver disease, lung disease, asthma, kidney disease, chronic haematological diseases, other chronic diseases and receiving immunosuppressive medicines had no association with COVID-19-related mortality. Underlying cardiovascular disease and neurological diseases were associated with COVID-19-related mortality (*P* < 0.023, *P* < 0.003 and *P* < 0.012, respectively). The presence of CT scan symptoms was significantly related to mortality in COVID-19 cases (*P* < 0.001). Having a risk factor was significantly correlated with mortality due to COVID-19 (*P* < 0.003). Having multiple risk factors was significantly correlated with mortality of COVID-19 (*P* < 0.002). The statistical test results presented above reveal the symptoms with significant relation to COVID-19 mortality. These symptoms can be used as features to form a decision tree for COVID-19 diagnosis. An example of these types of decision trees is shown in Fig. 3. The results of evaluating the prepared decision tree on our dataset are available in Table 2. The evaluation was done based on accuracy [41], sensitivity [42], specificity [43], precision [44] and F1-score [45]. In Fig. 4, the patients are shown according to their important features extracted by PCA, PSL and t-SNE algorithms. According to this figure, although PCA has better performance, it is clear that the cases are not separable well.

**Table 2:** The results of evaluating the decision tree on our dataset

Accuracy (%)	Precision (%)	Sensitivity (%)	Specificity (%)	F1-score (%)
87.41	90.64	88.69	82.32	89.21

## Discussion

The main findings of our study are the significant association of mortality due to COVID-19 with factors such as age, headache, chest pain, low respiratory rate, oxygen saturation less than 93%, need to a mechanical ventilator, having symptoms on CT, hospitalization in wards and time to infection. Besides, neurological disorders, cardiovascular diseases and having risk factor(s) were associated with COVID-19 mortality. Interestingly, there was no significant association between mortality and gender, fever, myalgia, dizziness, seizure, abdominal pain, nausea, vomiting, diarrhoea and anorexia. As another contribution, this paper is the first to investigate the association of history of neurological disorders, having risk factor(s), dizziness, seizure and abdominal pain with COVID-19-related mortality.

The significant association between age and COVID-19-related mortality in our study is in line with previous studies conducted by Zhou et al. [46], Pettit et al. [47], Chen et al.[48] and Iftime et al. [49] and in contrast to De Smet et al. [50], Sun et al. [51] and Li et al. [52]. Immune impairment and the enhanced possibility of developing cardiovascular and respiratory diseases would be the joint linkage between old age and COVID-19-related mortality [53, 54]. The observed association between the underlying cardiovascular diseases and COVID-19-related mortality in our study was in line with Chen et al. [55], Soares et al. [56] and Ruan et al. [57], but was contrary to Iftimie et al. [58], Li et al. [59] and Ciardullo et al. [60] findings. We found underlying high blood pressure to be associated with COVID-19 mortality, which is in line with Li et al. [59] finding and is in contrast with Rawl et al. [61], Pei et al. [62], Sun et al. [51] and Ciardullo et al. [60] findings. Hospitalization in wards was associated with COVID-19-related mortality, parallel with Chen et al. [59] findings, who found a relationship between ICU admission and mortality. The association between the need for mechanical ventilation and COVID-19-related mortality is in line with Chen et al. [59] and Zhou et al. [46] findings. The association between low oxygen saturation and low respiratory rate with mortality was in contrast with Sun et al. [51] findings.

In our previous study, anorexia, dry cough, anosmia and history of cancer were associated with COVID-19-related mortality [63], but in this study, we observed no relationship between mortality of COVID-19 and cancer that may be due to different populations of the study: two other provinces from one country. Anorexia showed a significant positive relationship with COVID-19-related mortality by Rawl et al. [61]. Regarding comorbidities, finding no significant association between cancer and COVID-19-related mortality is in line with Lee et al. [64] findings but is in contrast with Iftimie et al. [49], Mehta et al. [65], Dai et al. [66], Westblade et al. [67], Melo et al. [68] and Rüthrich et al. [69] findings. Different demographic features could explain this discrepancy. Finding no association between gender and COVID-19-related mortality is the same as Ruan et al. [57], Mehta et al. [65] and Sun et al. [51]. Absence of association between fever and COVID-19-related mortality in our study is the same as our previous research [63], but it contrasts with the findings of Iftime et al. [49]. Myalgia, diarrhoea, nausea and vomiting were not predictors of mortality in our cohort, which contrast with Zhou et al. [46] findings. Some of the typical clinical characteristics of COVID-19 patients with mortality was summarized in Table 3.

**Table 3:** Some of the typical clinical characteristics of COVID-19 patients with mortality

Reference	Study sample size	Country	Feature name	*P* value
Zhou et al. [46]	171	China	Acute cardiac injury	< 0.0001
			Acute kidney injury	< 0.0001
			Respiratory failure	< 0.0001
			Invasive/Non-invasive mechanical ventilation	< 0.0001
Pettit et al. [47]	238	USA	Age	< 0.0005
Li et al. [52]	269	China	Age, > _65 y vs < 65 y	0.021
Chen et al. [48]	1,590	China	Age (≥ 75 vs < 65)	< 0.001
De Smet et al. [50]	81	Belgium	Age	0.03
Iftime et al. [49]	188	Spain	Age	< 0.001
			Fever	0.046
Ruan et al. [57]	150	China	Gender	0.43
Reddy et al. [71]	47 Studies	Mixed nationalities	Smokers	< 0.0001
Magfira et al. [72]	Data from 74 countries	Mixed nationalities	Male smoking	0.16
Dai et al. [66]	641	China	Cancer	0.03
Mehta et al. [65]	218	United States	Cancer	< 2.2e-16
			Gender	0.6
Melo et al. [68]	60	Brazil	metastatic cancer	< 0.001
Lee et al. [64]	123	UK	Lung cancer	0.29
			Prostate cancer	0.82
			Leukaemia	0.023
Rüthrich et al. [69]	435	UK	Cancer	< 0.001
Westblade et al. [67]	2,914	USA	Hematologic malignancy	0.006
Chen et al. [59]	3309	China	Acute kidney injury	0.033
			Acute liver injury	< 0.0001
			Acute respiratory distress syndrome	< 0.0001
			Septic shock	< 0.0001
			Coagulation disorder	< 0.0001
			Oxygen treatment	0.390
			Mechanical ventilation	< 0.0001
			ICU admission	< 0.0001
			Systemic glucocorticoids	< 0.0001
Soares et al. [56]	10,713	Brazil	Kidney diseases	< 0.001
			Cardiovascular diseases	0.001
			Diabetes	0.003
			Obesity	< 0.001
			Smoking	< 0.001
			Race(Asian/indigenous/unknown)	< 0.001
			Shortness of breath	< 0.001
			Sore throat	< 0.001
Pei et al. [62]	198	China	Acute kidney injury	< 0.001
			Current pregnancy	< .0001
			History of solid organ transplant	0.2597
			History of chronic kidney disease	< 0.0001
			History of cardiovascular disease	< 0.0001
			History of hypertension	0.2716
Mendy et al. [73]	689	USA	smoker	0.659
			Diabetes	0.193
			Obesity	0.881
			Chronic kidney disease	0.001
			Anaemia	0.040
			Thrombocytopenia	< 0.001
			Coagulation defect	< 0.001
			Race/ethnicity (non-Hispanic Black)	0.012
Li et al. [59]	596	China	Hypertension	0.001
			Coronary heart disease	0.054
			Malignancy	0.120
Ciardullo et al. [60]	373	Italy	chronic obstructive pulmonary disease	0.084
			Cardiovascular diseases	0.348
			Hypertension	0.137
			Diabetes	0.253
Polon et al. [74]	57	Italy	Dementia	0.002
Sun et al. [51]	244	China	SpO2, %	0.565
			Respiratory rate, breaths/min	0.181
			Consciousness disorders (disorders vs clear)	0.827
			Hypertension	0.744
			Age	0.037
			Gender	0.270
Hue et al. [75]	74	France	Acute respiratory distress syndrome (ARDS) severity	0.007
Chen et al. [76]	145	China	Anorexia	0.01
Zhang et al. [77]	139	China	Anorexia	0.588
Homayounieh. [78]	90	Iran	Headache	0.3
			Chest pain	0.2
			Lower lung area	0.04
Sorouri et al. [79]			Fever	0.412
	172		Cough	0.398
			Chills	0.610
			Myalgia	0.990
			Nausea	0.135
			Diarrhoea	0.491
			Sore throat	0.990
			Fatigue	0.786
			Anorexia	0.076
			Chest pain	0.304
			Dyspnoea	0.013
Rawle et al. [61]	134	UK	Anorexia	0.028
			Respiratory disease	0.609
			Cardiac disease	0.333
			Diabetes mellitus	0.787
			Hypertension	0.728

The most important strength of this research is investigating impact of some new features on mortality rate of COVID-19 patients. Another important strength of this research is the large amount of data used. However, our results should be interpreted with the following weaknesses. The patients were recruited from a specific region, and our results might not apply in other countries as factors associated with mortality may differ in various regions [70]. Future research is necessary to investigate mortality rate of COVID-19 in patients with heart or kidney diseases with long-term follow-ups.

## Conclusion

In this research, we investigated the effect of some of the risk factors and symptoms of COVID-19 mortality rate for the first time. Our results show a significant association between mortality and risk factors like old age, headache, chest pain, low respiratory rate, oxygen saturation less than 93%, need to a mechanical ventilator, having symptoms on CT, hospitalization in wards, time to infection, neurological disorders, cardiovascular diseases and having a risk factor or multiple risk factors. In contrast, there is no significant association between mortality and gender, fever, myalgia, dizziness, seizure, abdominal pain, nausea, vomiting, diarrhoea and anorexia. More studies are needed to confirm these findings.

## Data Availability

The data that support the findings of this study are available on request from the corresponding author.
